# Supplementary material for: Characterization of a Novel POx-Based Adhesive Powder for Obliterating Dead Spaces After Surgery
Source: Bioengineering (Basel). 2025 Sep 23;12(10):1011. doi: 10.3390/bioengineering12101011 (PMC12561639; doi:10.3390/bioengineering12101011)
Supplement: Supplementary file 1 [file bioengineering-12-01011-s001.zip › bioengineering-3820508-supplementary.pdf]

bioengineering

MDPI

Supplementary Material

Characterization of a Novel POx-Based Adhesive Powder for Obliterating Dead Spaces After Surgery

Steven E. M. Poos [1,2,*], Roger M. L. M. Lomme [1], Edwin A. Roozen [2], Johan C. M. E. Bender [2], Harry van Goor [1] and Richard P. G. Ten Broek [1]

 https://doi.org/10.3390/bioengineering12101011

bioengineering

SA Schematic production of all powder prototypes used in the current study

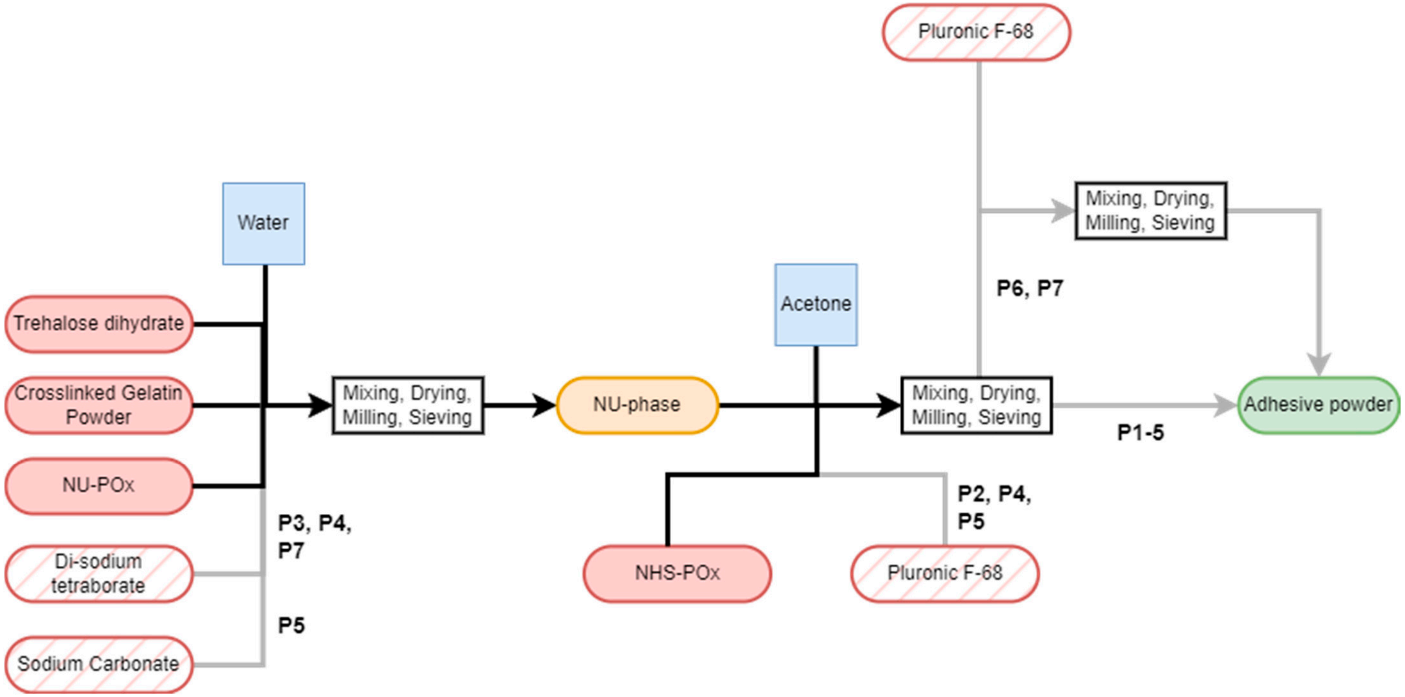

Figure S1. Production process of adhesive powder prototypes. Black arrows indicate steps performed for every prototype; grey arrows indicate steps performed for distinct prototypes mentioned next to the arrow. Red curved boxes indicate resources, where a solid fill indicates a resource used in all prototypes and a hatched fill indicates a resource used for distinct prototypes. Blue squares indicate solvents used. White squares with black borders indicate production steps. The orange curved box indicates an intermediate product, i.e., NU-phase. The green box indicates the finished product, i.e., adhesive powder.

SB Chemical characterization of adhesive powder prototypes

pH measurement

pH has an influence on both adhesion and stability, and is, therefore, an important characteristic. During production process, the pH of intermediate NU phase is measured by adding 5 mL dH20 to 100 mg and determine pH values via pH measuring rod, while pH of the powder is measured by adding 1 ml dH2O to 100 mg powder. Powder pH measuring process starts by adding 100 mg of powder in a 10 mL falcon tube. The powder-containing tube is placed on a vortex and 1 mL of dH2O is added. While the substance is still a liquid, the pH is measured by use of a pH strip (VWR, Radnor, PA, USA).

Active group degree of functionality (NHS-DF) measurement

Nuclear Magnetic Resonance (NMR) analysis is performed to measure the degree of NHS functionality (NHS-DF); the percentage of NHS-ester groups still present on NHS-POx polymers. Samples are created by first deactivating NU-POx polymers in an extraction solution from benzoic anhydride (Bz_2O, Thermo-Fisher Scientific, Waltham, MA, USA) and chloroform-d_1(VWR, Radnor, PA, USA). Then, insoluble crosslinked gelatine is filtered out using a 0.22 μm nylon filter(VWR, Radnor, PA, USA). Subsequently, the sample is dissolved in DMSO-d_6 (Deutero GmbH, Kastellaun, Germany) and inserted to NMR tubes (Deutero GmbH, Kastellaun, Germany) for ^{1}H-NMR-spectrum analysis (JEOL, Tokyo, Japan). The achieved NMR spectra are processed in MestReNova (Mestrelab Research, Barcelona, Spain), where the peak intensity at ± 2.8 ppm relative to the peak intensity at ± 4 ppm is used to determine the NHS-DF percentages.

Water Content measurement

Water content of the powder is measured in percentages via a Karl Fisher colorant assay. Powder sample is mixed with hydranal (Honeywell, Charlotte, NC, USA) mixture and added to a hydranal chamber in a Karl Fisher (Mettler Toledo, Greifensee, Switzerland) to determine water content via coulometric titration to determine water traces in the sample. The equipment is validated using an internal standard, sodium tartare dihydrate (VWR, Radnor, PA, USA), before and after sample measurement.

 https://doi.org/10.3390/bioengineering12101011

SC Chemical specifics powder prototypes

Prototype	LOT powder	Buffer	Pluronic	Particle size	pH	NHS-DF	Water content
P1	P240201	X	X	125-250	4,5	69	1,24
P1	P231002	X	X	125-250	4,5	113,8	4,52
P1	P240101	X	X	125-250	4,5	101,1	4,72
P1	P240601	X	X	250-500	4,5	85,2	4,98
P1	P231001	X	X	250-500	4,5	113,8	4,52
P2	P230803	X	Impreg	125-250	4,5	94,5	9,51
P2	P231006	X	Impreg	125-250	4,5	84,1	6,64
P2	P240603	X	Impreg	250-500	4,5	95,3	5,63
P2	P231005	X	Impreg	250-500	4,5	84,1	6,64
P3	P240405	Borax	X	125-250	7,5	111,1	5,26
P3	P231008	Borax	X	125-250	7,5	64,3	5,45
P3	P240405	Borax	X	250-500	7,5	111,1	5,26
P3	P231007	Borax	X	250-500	7,5	64,3	5,45
P4	P240404	Borax	Impreg	125-250	7,5	122,3	5,17
P4	P231012	Borax	Impreg	125-250	7,5	71,1	6,25
P4	P240404	Borax	Impreg	250-500	7,5	122,3	5,17
P4	P231011	Borax	Impreg	250-500	7,5	71,1	6,25
P5	P240305	Carb	Impreg	125-250	6,5	97,9	3,38
P5	P240401	Carb	Impreg	125-250	6,5	103,7	3,48
P5	P240305	Carb	Impreg	250-500	6,5	97,9	3,38
P5	P240401	Carb	Impreg	250-500	6,5	103,7	3,48
P6	P230804	X	Coat	125-250	4,5	81	6,12
P6	P240306	X	Coat	125-250	4,5	324,8	3,73
P6	P240302	X	Coat	125-250	4,5	91,7	3,32
P6	P240903	X	Coat	250-500	4,5	81,5	4,85
P7	P231010	Borax	Coat	125-250	7,5	73,6	5,27
P7	P231009	Borax	Coat	250-500	7,5	73,6	5,27

SD Internal calibration curves for *in vitro* degradation measurement

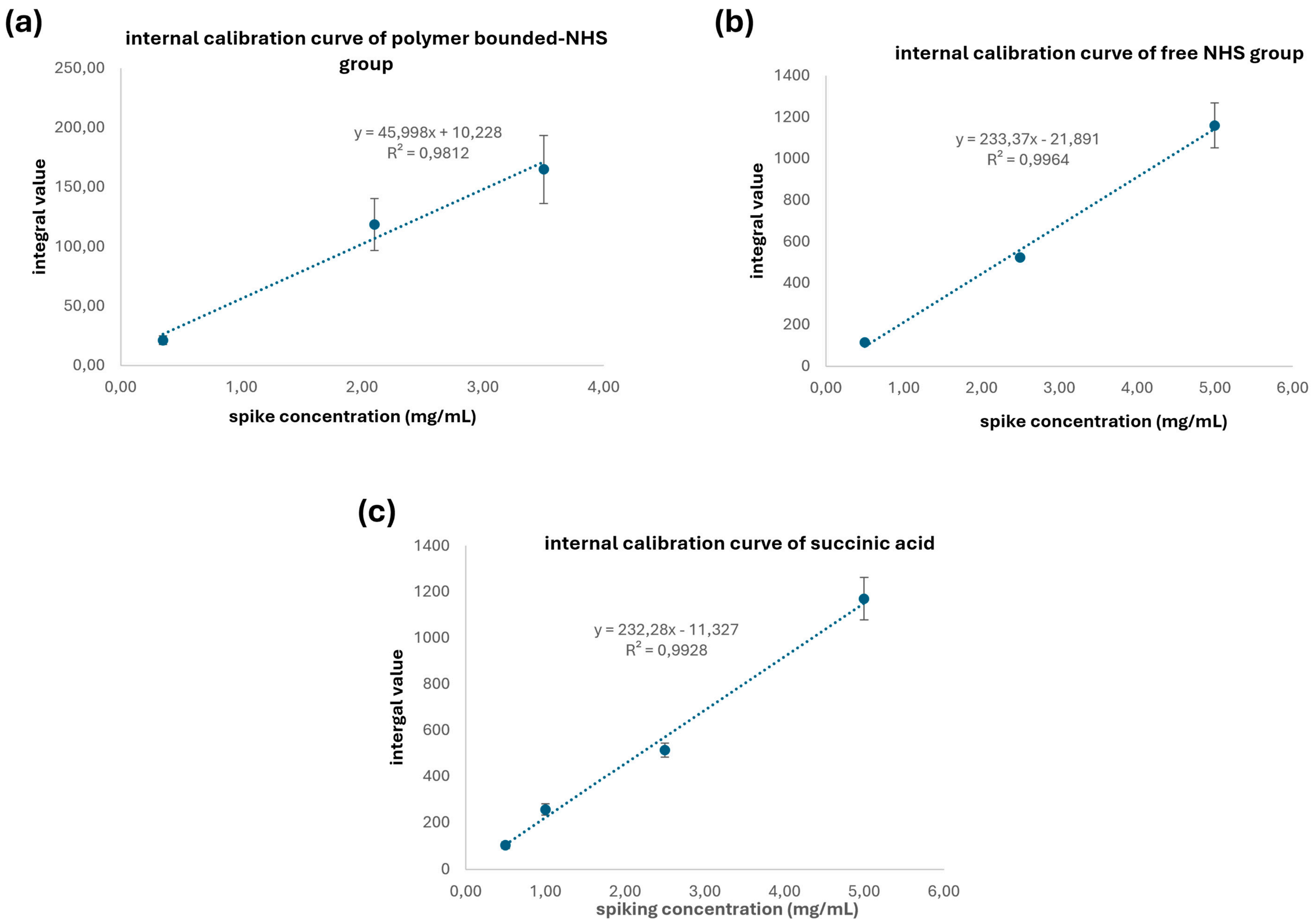

Figure S2. Wetting curves of ingredients used in every adhesive powder formulation (i.e., Trehalose, crosslinked gelatin, NU-POx, and NHS-POx), intermediate product NU-phase and a positive control (silica gel) and negative control (PET tablets). Number of repetitions is indicated in the legend for every tested group. PC = positive control, NC = negative control.

https://doi.org/10.3390/bioengineering12101011

bioengineering MDPI

SE Wettability ingredients and intermediate products

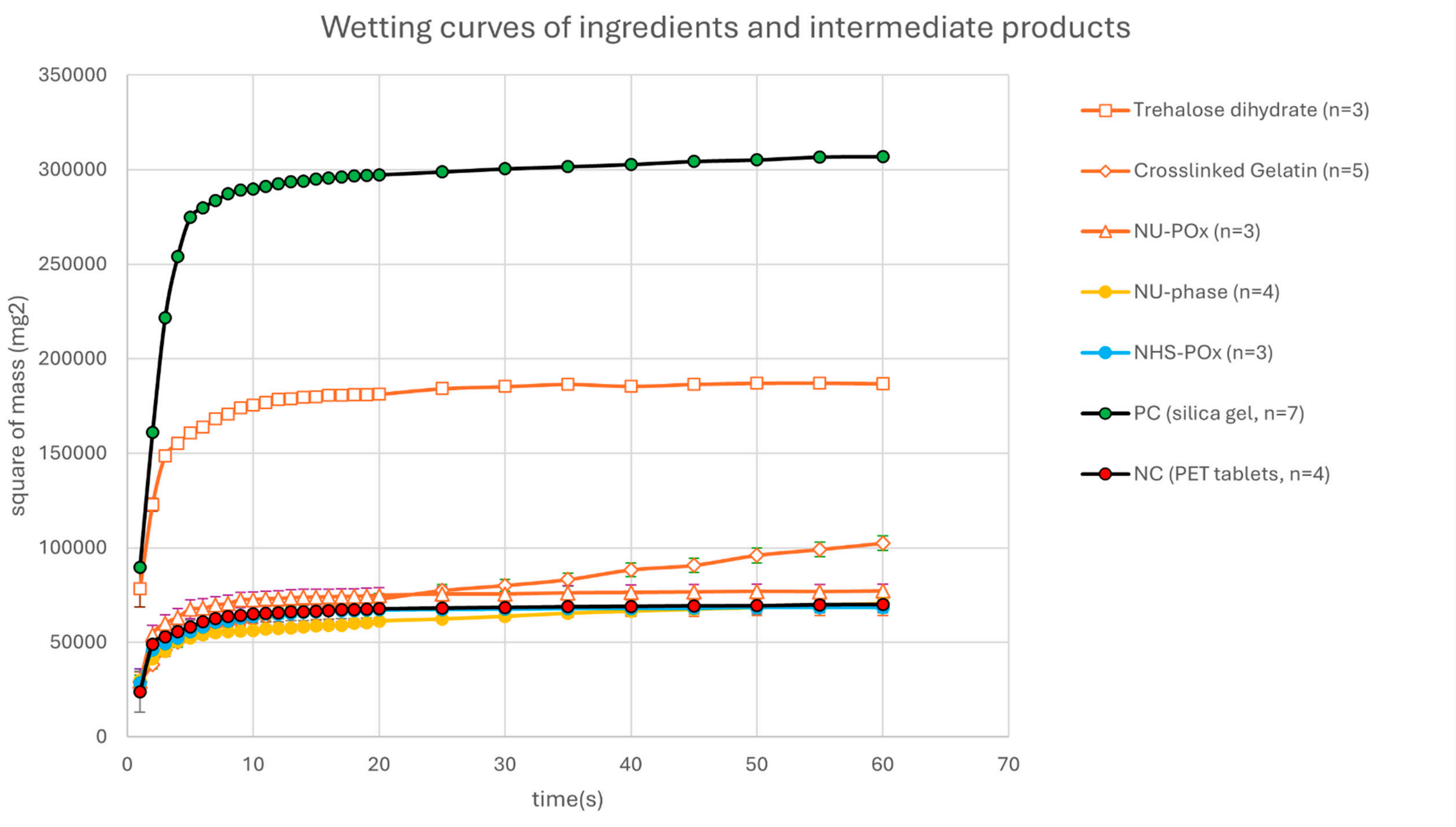

Figure S3. Wetting curves of ingredients used in every adhesive powder formulation (i.e., Trehalose, crosslinked gelatin, NU-POx, and NHS-POx), intermediate product NU-phase and a positive control (silica gel) and negative control (PET tablets). Number of repetitions is indicated in the legend for every tested group. PC = positive control, NC = negative control.

SF Swelling experiment with pictures

	t_{00}	t_{0}	t_{2h}	t_{24h}
Adhesive powder (125-250 µm)	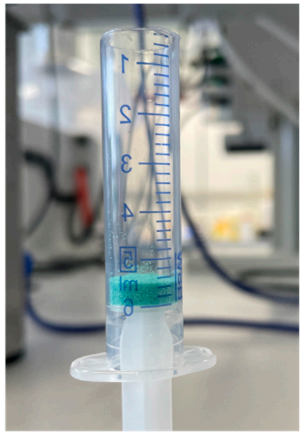	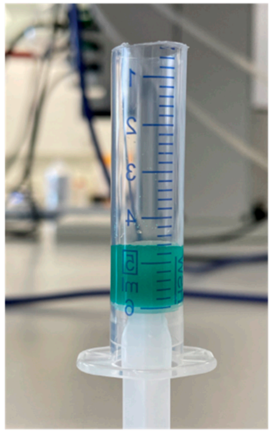	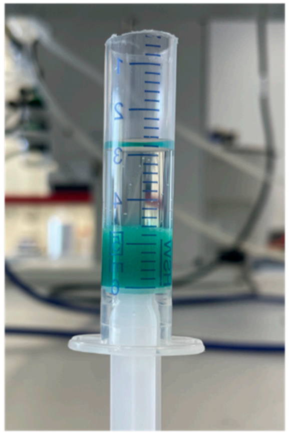	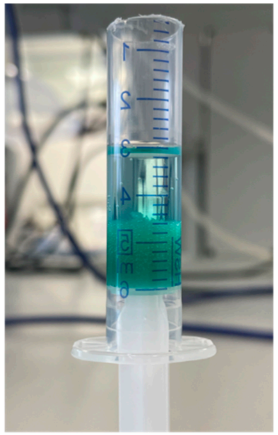
Crosslinked gelatin powder	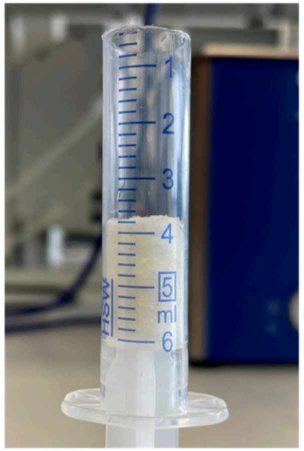	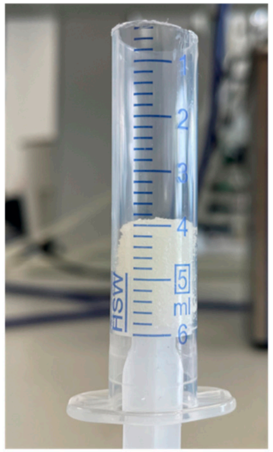	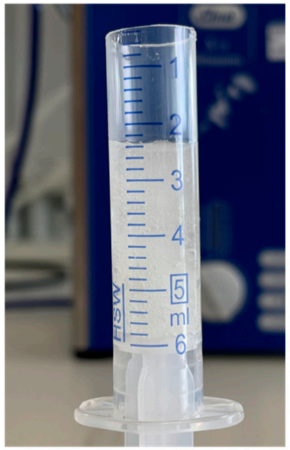	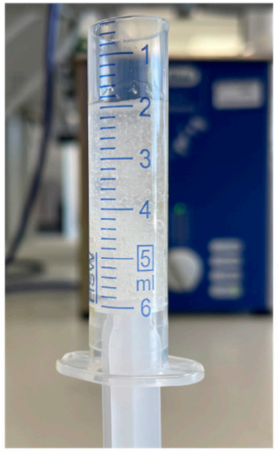

SG Degradation of adhesive powder per byproduct

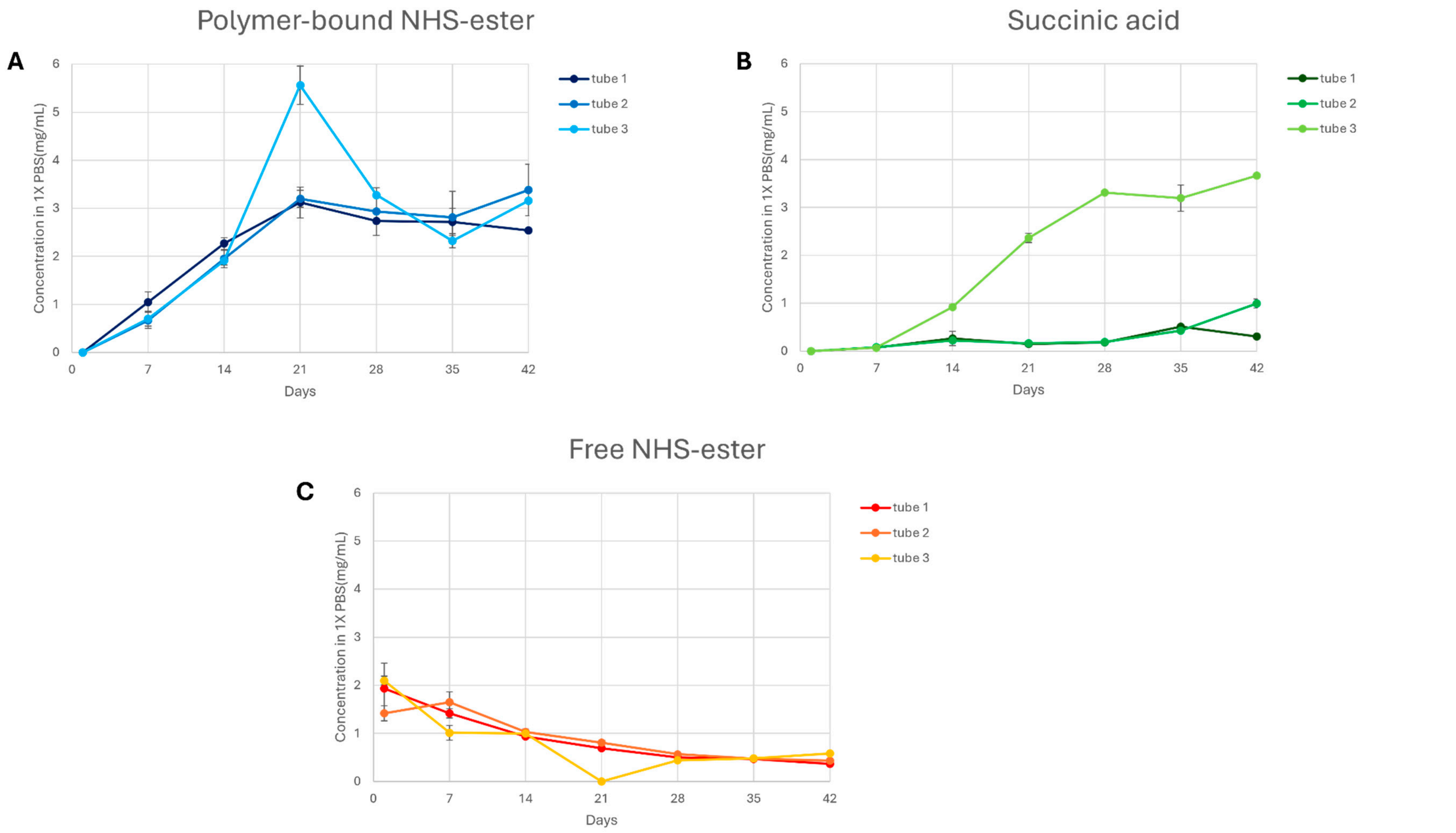

bioengineering

MDPI

SH P-values for wettability outcomes

Table S1. P values of ANOVA and Tukey HSD Post-hoc analysis between the total water uptake of the different tested prototypes during Washburn capillary rise tests.

	P1 s	P1 l	P2 s	P2 l	P3 s	P3 l	P4 s	P4 l	P5 s	P5 l	P6 s	P6 l	P7 s	P7 l
P1 s	1	0.0172355634153572	0.0583947279852824	0.00662343571891361	0.149555864099491	0.145905044024907	0.00709610752717218	0.199874821917553	0.0201873902509912	0.0507026029460908	0.133871260475074	0.0355685166606582	0.151084533219975	0.284097559776205
P1 l	0.0172355634153572	1	0.0892755179931564	0.966357639398896	0.154810538830002	0.264567316745733	0.431850011920871	0.860097071617373	0.766781259317764	0.753347651245674	0.062307196928473	0.886215201860236	0.234539174737088	0.878946152326546
P2 s	0.0583947279852824	0.0892755179931564	1	0.029030779497893	0.949646394139747	0.828250985774158	0.04037677926516	0.430010450903566	0.0883274248956698	0.177822014091792	0.543772838954077	0.152724354255242	0.884912870735423	0.613442894041585
P2 l	0.00662343571891361	0.966357639398896	0.029030779497893	1	0.0984826307625006	0.202882287351778	0.189952015399361	0.867000889698077	0.756154249308897	0.752314071978185	0.0230345172740281	0.89609390010501	0.174110419289274	0.864982425804596
P3 s	0.149555864099491	0.154810538830002	0.949646394139747	0.0984826307625006	1	0.822490524601805	0.1973883616687	0.433543295800127	0.134387537777817	0.206601134673313	0.729568167740346	0.196285869901939	0.869656302361319	0.611648506879497
P3 l	0.145905044024907	0.264567316745733	0.828250985774158	0.202882287351778	0.822490524601805	1	0.395870405418146	0.498171973049687	0.217419859556506	0.282922057194811	0.585575880834929	0.290329320439787	0.952631524352933	0.692105748223361
P4 s	0.00709610752717218	0.431850011920871	0.04037677926516	0.189952015399361	0.1973883616687	0.395870405418146	1	0.68424551251659	0.329542578948391	0.449086737577743	0.0324364546110232	0.483884858665417	0.343226505565734	0.936205162955985
P4 l	0.199874821917553	0.860097071617373	0.430010450903566	0.867000889698077	0.433543295800127	0.498171973049687	0.68424551251659	1	0.960477268560446	0.996641894164788	0.364651487023964	0.917276926936837	0.480078108335204	0.809483205455546
P5 s	0.0201873902509912	0.766781259317764	0.0883274248956698	0.756154249308897	0.134387537777817	0.217419859556506	0.329542578948391	0.960477268560446	1	0.927525372233949	0.0638220765268016	0.910662297679067	0.194603029772843	0.782008784836669
P5 l	0.0507026029460908	0.753347651245674	0.177822014091792	0.752314071978185	0.206601134673313	0.282922057194811	0.449086737577743	0.996641894164788	0.927525372233949	1	0.134241665387322	0.861083939653745	0.261884201299937	0.757310037693701
P6 s	0.133871260475074	0.062307196928473	0.543772838954077	0.0230345172740281	0.729568167740346	0.585575880834929	0.0324364546110232	0.364651487023964	0.0638220765268016	0.134241665387322	1	0.110014698884026	0.625393598328091	0.522487699339788
P6 l	0.0355685166606582	0.886215201860236	0.152724354255242	0.89609390010501	0.196285869901939	0.290329320439787	0.483884858665417	0.917276926936837	0.910662297679067	0.861083939653745	0.110014698884026	1	0.264517680572463	0.83120756652255
P7 s	0.151084533219975	0.234539174737088	0.884912870735423	0.174110419289274	0.869656302361319	0.952631524352933	0.343226505565734	0.480078108335204	0.194603029772843	0.261884201299937	0.625393598328091	0.264517680572463	1	0.669583494927149
P7 l	0.284097559776205	0.878946152326546	0.613442894041585	0.864982425804596	0.611648506879497	0.692105748223361	0.936205162955985	0.809483205455546	0.782008784836669	0.757310037693701	0.522487699339788	0.83120756652255	0.669583494927149	1

Table S2. P values of ANOVA and Tukey HSD Post-hoc analysis of the average time until a plateau in water uptake was reached between the tested prototypes during Washburn capillary rise tests.

	**P1 s**	**P1 l**	**P2 s**	**P2 l**	**P3 s**	**P3 l**	**P4 s**	**P4 l**	**P5 s**	**P5 l**	**P6 s**	**P6 l**	**P7 s**	**P7 l**
**P1 s**	1	0.22153105880923	0.178791181705843	0.180077069133423	0.208546950551783	0.410949263587831	0.425021114248696	0.453410526661471	0.0240437591073604	0.237620399114144	0.178791181705843	0.207483470220608	1	0.592843364485601
**P1 l**	0.22153105880923	1	0.0764639094151493	0.770977332327307	0.613328175359967	0.602607442575666	0.438198678198331	0.67749124902012	0.104688700574837	0.622268580833678	0.0764639094151493	0.966338143210904	0.293920024968364	0.946874693701648
**P2 s**	0.178791181705843	0.0764639094151493	1	0.0418476430798322	0.107172481475946	0.114873407958949	0.0785111986906075	0.344013261842586	0.0133910921607661	0.0483589200350324	0.999999999999999	0.066690704565917	0.342826736997225	0.370656684149594
**P2 l**	0.180077069133423	0.770977332327307	0.0418476430798322	1	0.471039446655208	0.738484512585089	0.491767001022169	0.617307367293323	0.0600481318796749	0.781025266608817	0.0418476430798322	0.795255008453841	0.30752735264957	0.944145705594372
**P3 s**	0.208546950551783	0.613328175359967	0.107172481475946	0.471039446655208	1	0.394780719828645	0.317078730869602	0.861274219691452	0.324769953332326	0.40157455385107	0.107172481475946	0.586724890297296	0.237269065939213	0.685968508115468
**P3 l**	0.410949263587831	0.602607442575666	0.114873407958949	0.738484512585089	0.394780719828645	1	0.814902011459181	0.568117487621546	0.055682985289601	0.907623266959919	0.114873407958949	0.614139550186501	0.509185568403459	0.837893461675563
**P4 s**	0.425021114248696	0.438198678198331	0.0785111986906075	0.491767001022169	0.317078730869602	0.814902011459181	1	0.531007362567313	0.0375901115111464	0.656045011943182	0.0785111986906074	0.435330942514376	0.574307058777486	0.761039785546133
**P4 l**	0.453410526661471	0.67749124902012	0.344013261842586	0.617307367293323	0.861274219691452	0.568117487621546	0.531007362567313	1	0.730997095396232	0.581469065581353	0.344013261842586	0.666860807005239	0.459347749522922	0.683302690226119
**P5 s**	0.0240437591073604	0.104688700574837	0.0133910921607661	0.0600481318796749	0.324769953332326	0.055682985289601	0.0375901115111464	0.730997095396232	1	0.0486658536334501	0.0133910921607661	0.0935990209703679	0.033787429587045	0.242161597299581
**P5 l**	0.237620399114144	0.622268580833678	0.0483589200350324	0.781025266608817	0.40157455385107	0.907623266959919	0.656045011943182	0.581469065581353	0.0486658536334501	1	0.0483589200350324	0.634027161196277	0.388482983953373	0.869580725188324
**P6 s**	0.178791181705843	0.0764639094151493	0.999999999999999	0.0418476430798322	0.107172481475946	0.114873407958949	0.0785111986906074	0.344013261842586	0.0133910921607661	0.0483589200350324	1	0.0666907045659171	0.342826736997225	0.370656684149594
**P6 l**	0.207483470220608	0.966338143210904	0.066690704565917	0.795255008453841	0.586724890297296	0.614139550186501	0.435330942514376	0.666860807005239	0.0935990209703679	0.634027161196277	0.0666907045659171	1	0.288526201758003	0.964098666862377
**P7 s**	1	0.293920024968364	0.342826736997225	0.30752735264957	0.237269065939213	0.509185568403459	0.574307058777486	0.459347749522922	0.0337874295587045	0.388482983953373	0.342826736997225	0.288526201758003	1	0.607922313499632
**P7 l**	0.592843364485601	0.946874693701648	0.370656684149594	0.944145705594372	0.685968508115468	0.837893461675563	0.761039785546133	0.683302690226119	0.242161597299581	0.869580725188324	0.370656684149594	0.964098666862377	0.607922313499632	1

SI Pictures of samples after Washburn test

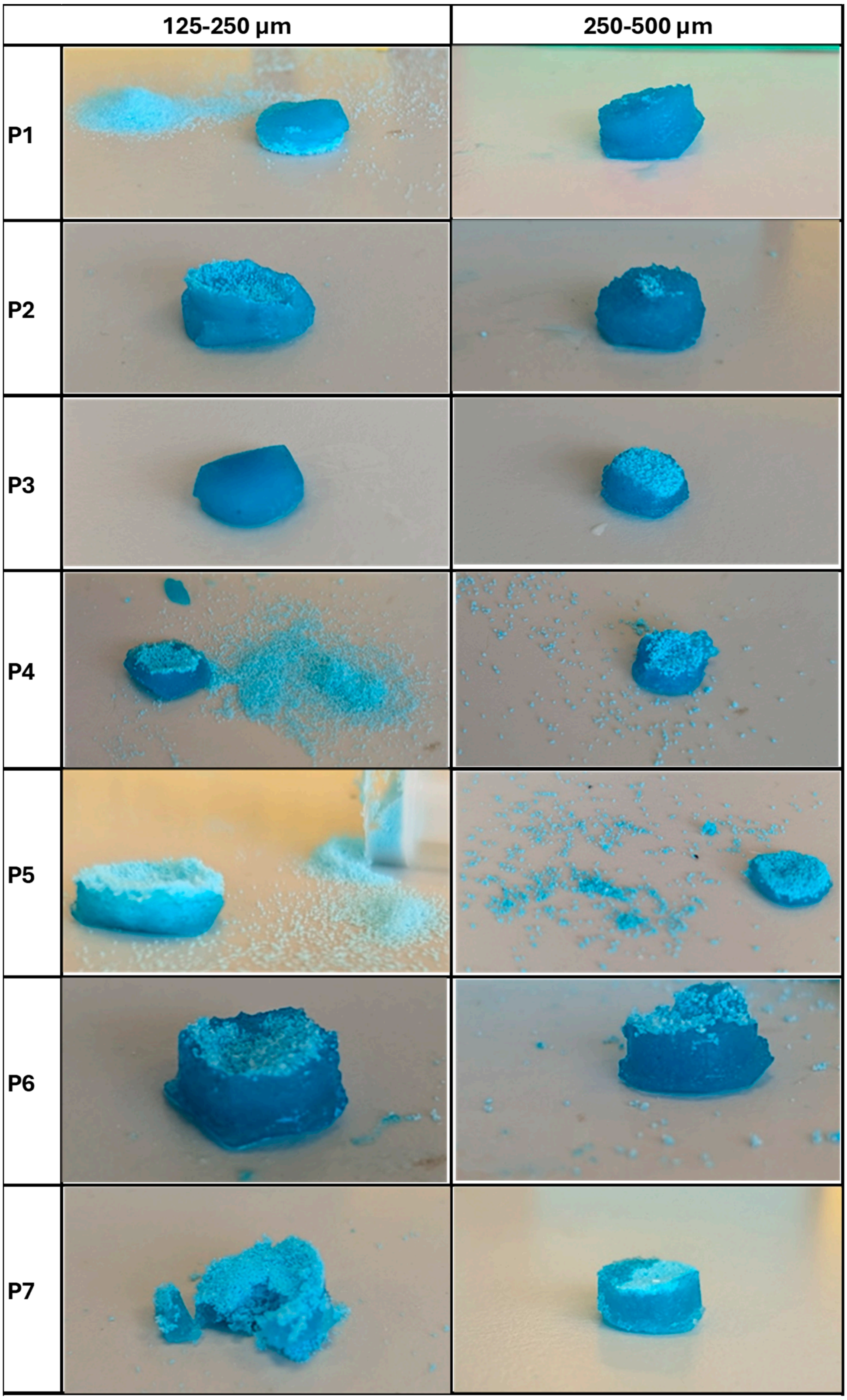

Figure S4. Photos of power prototypes **P1–7** after Washburn capillary test performance. Both smaller (125–250 µm) and larger (250–500 µm) particle size are displayed.
